# Movable Kidney and Hepatic Colic

**Published:** 1901-02-02

**Authors:** 


### MOVABLE KIDNEY AND HEPATIC COLIC.

The observation which has several times been made of late, that a movable kidney may set up symptoms strongly suggestive of, even if they do not really indicate the presence of hepatic colic, is one which must always be borne in mind in investigating these diseases. Dr. Macpherson Lawrie[1] has recorded another case of this nature. The patient had been in bad health for several years, many of the symptoms suggesting the presence of gall stones, and while under observation she developed an attack of jaundice with much pain and other symptoms, all pointing in the same direction. On examination the right kidney was found to be freely movable. Operating through a lumbar incision the kidney was stitched to the deep muscles of the back, and after this the patient rapidly improved and lost her attacks. It is to be presumed that in these cases, in addition to the distress caused by the proptosis of the kidney, others are added, due to its occasional impaction in such a position as to interfere with the due patency of the hepatic or common duct.

[1] Brit. Med. Journ., Jan. 5.

